# Gestational diabetes and risk of type 2 diabetes: exploring the role of the gut microbiome in the Hispanic Community Health Study/Study of Latinos (HCHS/SOL)

**DOI:** 10.1007/s00125-026-06727-0

**Published:** 2026-04-30

**Authors:** Yi Wang, Carmen R. Isasi, Alison M. Stuebe, Adetola F. Louis-Jacques, Jie Hu, Gang Hu, Martha L. Daviglus, Eric Boerwinkle, Robert D. Burk, Robert C. Kaplan, Qibin Qi, Brandilyn A. Peters

**Affiliations:** 1 Department of Epidemiology and Population Health, Albert Einstein College of Medicine, Bronx, NY USA; 2 Division of Maternal Fetal Medicine, Department of Obstetrics and Gynecology, University of North Carolina Chapel Hill, Chapel Hill, NC USA; 3 Department of Obstetrics and Gynecology, University of Florida College of Medicine, Gainesville, FL USA; 4 Department of Epidemiology, Harvard T.H. Chan School of Public Health, Boston, MA USA; 5 Chronic Disease Epidemiology Laboratory, Pennington Biomedical Research Center, Baton Rouge, LA USA; 6 Institute for Minority Health Research, University of Illinois Chicago, Chicago, IL USA; 7 Department of Epidemiology, Human Genetics, and Environmental Sciences, School of Public Health, University of Texas Health Science Center at Houston, Houston, TX USA; 8 Departments of Pediatrics, Albert Einstein College of Medicine, Bronx, NY USA; 9 Department of Microbiology & Immunology, Albert Einstein College of Medicine, Bronx, NY USA; 10 Department of Obstetrics, Gynecology & Women’s Health, Albert Einstein College of Medicine, Bronx, NY USA; 11 Division of Public Health Sciences, Fred Hutchinson Cancer Research Center, Seattle, WA USA

**Keywords:** Gestational diabetes, Gut microbiome, Metabolomics, Type 2 diabetes

## Abstract

**Aims/hypothesis:**

Women with a history of gestational diabetes mellitus (GDM) have an elevated risk of type 2 diabetes in their later life, yet the underlying mechanisms of this remain unclear. We aimed to investigate the long-term impact of GDM on gut microbiota and related metabolites and to explore whether such alterations may contribute to type 2 diabetes risk.

**Methods:**

Among parous women from the Hispanic Community Health Study/Study of Latinos (HCHS/SOL), we identified microbial species associated with a history of GDM (visit 2, 2014–2017, *n*=1525), and serum metabolites associated with both a history of GDM (visit 1, 2008–2011, *n*=2968) and GDM-related microbiota (visit 2, *n*=391). We further examined prospective associations of the GDM-related microbiome (visit 2, *n*=925) with incident type 2 diabetes over 6 years of follow-up, and of microbial-related metabolites (visit 1, *n*=2341) with incident type 2 diabetes over 12 years.

**Results:**

Among 1525 US Hispanic/Latino parous women (median age: 58 years), seven species differed between women with and without a history of GDM, including higher abundances of four species (e.g. *Parabacteroides merdae CAG:48*, a proinflammatory taxon) and lower abundances of three species (e.g. *Dialister sp. CAG:588*, a short-chain fatty acid producer). Fifteen metabolites were associated with both a history of GDM and the GDM-related microbiome in a consistent direction, nine of which (e.g. saturated sphingomyelins and unsaturated fatty acids) were associated with glycaemic traits and incident type 2 diabetes. Using these microbial-related metabolites as proxy measures, proxy analysis suggested a positive relationship between the GDM-related microbiome and type 2 diabetes (*r*=0.55, *p*=0.036). A metabolite score derived from the nine microbial-related metabolites mediated an estimated 20% (95% CI 9%, 42%) of the association between a history of GDM and type 2 diabetes.

**Conclusions/interpretation:**

A history of GDM is associated with an unfavourable gut microbiota and related metabolites in US Hispanic/Latino women, suggesting a potential role of the gut microbiota in GDM-related type 2 diabetes.

**Graphical Abstract:**

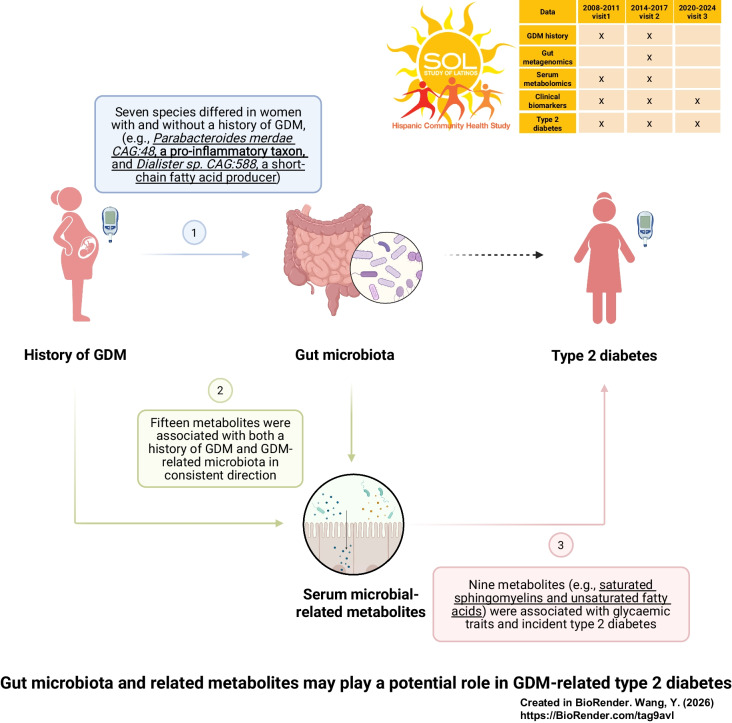

**Supplementary Information:**

The online version contains peer-reviewed but unedited supplementary material available at 10.1007/s00125-026-06727-0.


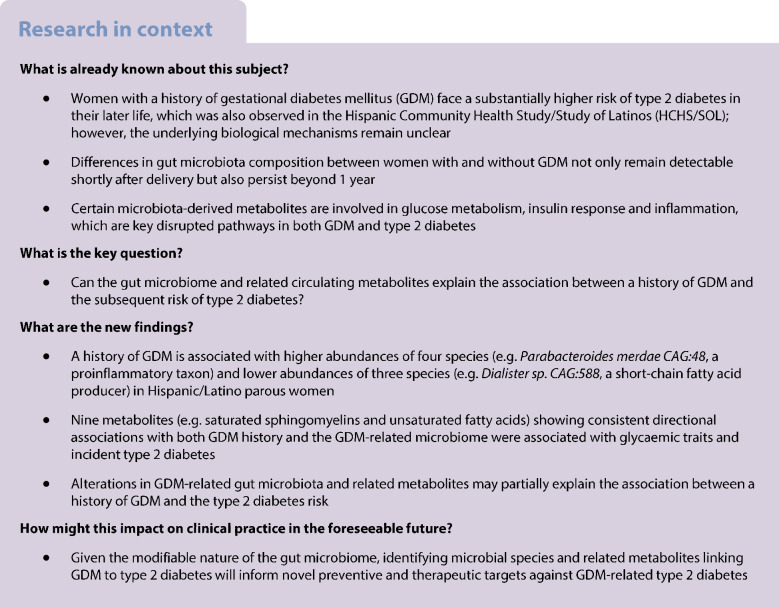


## Introduction

Gestational diabetes mellitus (GDM) is the most common complication of pregnancy affecting approximately 5–9% of pregnancies in the USA every year [1]. Although GDM is typically resolved shortly after delivery, women with a history of GDM face a substantially higher risk of developing type 2 diabetes than those with normoglycaemic pregnancy [2], which was also observed in the Hispanic Community Health Study/Study of Latinos (HCHS/SOL) [3], a population with higher birth rates [4] and a disproportionately higher burden of diabetes than non-Hispanic white populations [3]. However, the molecular mechanisms underlying progression from GDM to type 2 diabetes remain incompletely understood.

Emerging evidence demonstrates that the gut microbiota and its microbiome-derived metabolites are implicated in glucose metabolism and insulin sensitivity, thereby playing a critical role in maintaining glucose homeostasis [5]. Several epidemiological studies have revealed distinct gut microbiota dysbiosis in women with GDM compared with those with normoglycaemia during the same trimester of pregnancy [6–12], with this dysbiosis persisting for over 1 year postpartum [10, 13–16]. Notably, the gut microbiota dysbiosis observed in people with GDM closely resembles that of individuals with type 2 diabetes [13, 17], particularly with respect to the reduced abundance of short-chain fatty acid (SCFA)-producing bacteria and an increased abundance of proinflammatory bacteria.

Metabolomics studies have revealed metabolic shifts in women with prior GDM that are associated with an increased risk of type 2 diabetes [18], including perturbations of some microbiota-derived metabolites involved in the regulation of host glucose metabolism, insulin response and inflammation [6, 7]. However, it remains largely unclear how GDM may affect the gut microbiome and related metabolites in the long term and, more importantly, how these potential alterations may contribute to the subsequent development of type 2 diabetes.

To address this gap, we leveraged stool shotgun metagenomics and serum metabolomics data in the HCHS/SOL to: (1) identify differential gut microbial species between women with and without a history of GDM; (2) identify circulating metabolites correlated with GDM-related species, under the premise that microbiota-derived metabolites could serve as functional intermediators between the gut microbiome and host biology; and (3) investigate the associations of these selected microbial species and circulating metabolites with incident type 2 diabetes and metabolic traits.

## Methods

The HCHS/SOL is a prospective population-based cohort study comprising 16,415 self-identified Hispanic/Latino adults (~60% women) aged 18–74 years from four US field centres (Bronx, NY; Chicago, IL; Miami, FL; and San Diego, CA, USA), as previously described [19]. The baseline visit (visit 1) was performed in 2008–2011, the second visit (visit 2) was performed in 2014–2017 and the third visit (visit 3) was performed in 2020–2024. At all visits, participants completed interviewer-administered questionnaires, provided biological specimens and underwent comprehensive physical examinations. The HCHS/SOL Gut Origins of Latino Diabetes ancillary study collected stool samples from 3035 participants approximately concurrent with visit 2. The present study was restricted to women with a history of pregnancy, because GDM occurs only among women who have been pregnant. The analytic samples were further limited to participants with available gut microbiome or metabolomics data. As a result, the final analytic samples represent subsets of the larger HCHS/SOL cohort. Although participants included in the omics analyses generally reflect the demographic characteristics of the underlying cohort (i.e. parous women) in terms of age distribution, Hispanic/Latino background and socioeconomic factors, the availability of omics data may introduce some selection and therefore the analytic samples may not be fully representative of the entire HCHS/SOL cohort.

**Fig. 1:**
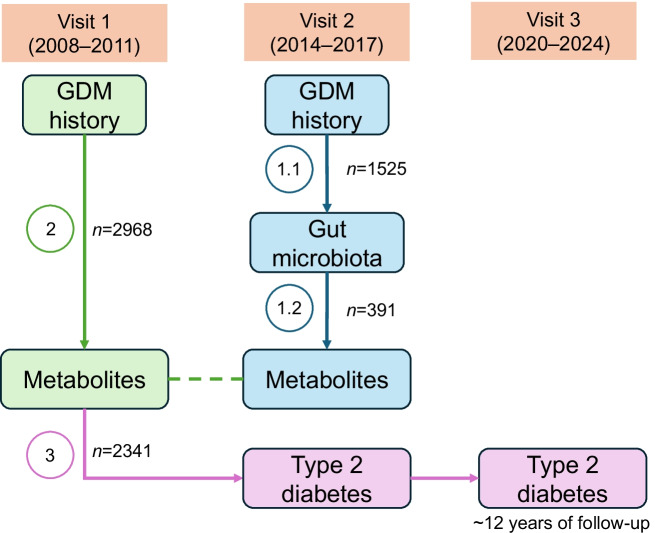
Overview of participant selection and data integration in the HCHS/SOL. The figure illustrates the multi-step analytical framework, with colour-coded boxes and arrows representing three major steps: (1) identification of the gut microbial species associated with a history of GDM, followed by the identification of serum metabolites correlated with these GDM-related species (blue); (2) selection of serum metabolites that were both associated with a history of GDM and directionally consistent with GDM-related gut microbiome (green); and (3) prospective evaluation of the associations between selected serum metabolites and incident type 2 diabetes (purple). At visit 1, 248 (8.4%) of 2968 women had a history of GDM; at visit 2, 168 (11.0%) of 1525 women had a history of GDM

Sample size varied across different analyses, depending on the availability of omics data for each integrative analysis (Fig. 1 and electronic supplementary material [ESM] Methods). Specifically, a total of 8780 women had a history of pregnancy prior to visit 2. After excluding women with missing data on their GDM history or gut microbiome at visit 2, those with <100,000 sequence reads in their metagenome sample and those with prevalent cardiovascular disease or cancer, a total of 1525 women were included in the analysis examining the association between GDM history and the gut microbiome. Among these women, 391 with concurrent serum metabolites data at visit 2 were included in the analysis examining the metabolites correlated with identified GDM-related microbiota. Furthermore, a total of 8765 women reported having been pregnant prior to visit 1. After excluding those with missing data on their GDM history or serum metabolomics at visit 1, as well as those with prevalent cardiovascular disease or cancer, a total of 2968 parous women were included in the analysis examining the association between GDM history and serum metabolites. Among these women, 2341 without prevalent type 2 diabetes at visit 1 were included in the analysis examining the prospective associations of metabolites with incident type 2 diabetes. The study was approved by the Institutional Review Boards of all study sites. All participants provided written informed consent.

### GDM history assessment

The history of GDM, defined as any degree of glucose intolerance that first occurred during pregnancy, was self-reported by women at each visit. Specifically, a history of GDM prior to visit 1 was ascertained by answering ‘yes’ to either of the following: (1) a physician diagnosis of diabetes only during pregnancy reported in the visit 1 questionnaire; or (2) a repeated follow-up question at visit 2 asking for diabetes first diagnosed during pregnancy prior to visit 1 (80% of women participated in both visit 1 and visit 2). In addition, a history of GDM prior to visit 2 was ascertained by the sum of women reporting GDM prior to visit 1 and those who reported a new GDM diagnosis occurring between visit 1 and visit 2.

### Gut metagenomic sequencing and bioinformatics processing

Shotgun metagenomics sequencing was conducted in the Knight Lab at the University of California San Diego, La Jolla, CA, USA [20]. DNA was extracted from stool samples following the Earth Microbiome Project protocol [21]. This protocol incorporates rigorous contamination control, standardised mechanical and chemical lysis, sequential inhibitor removal, and membrane-based spin filter purification with ethanol-containing wash steps to ensure high-quality DNA suitable for downstream sequencing. Extracted DNA was quantified and normalised, and an automated workflow was performed for the library preparation. The sequencing adapters and barcode indices were added following the iTru adapter protocol [22]. The final indexed libraries were purified, quantified, normalised and pooled for sequencing on the Illumina NovaSeq 6000 platform (San Diego, CA, USA). Raw FASTQ sequence reads were processed using the standard shotgun sequencing pipeline implemented in *Qiita* [23]*.* Per-sample sequence adapters were trimmed via *fastp* [24], and sequence reads mapping to the human reference genome (GRCh38) were identified and removed using *minimap2* (qp-fastp-minimap2 2022.04) [25]. The sequence reads were then aligned against the WolR1 [26] reference database of bacterial and archaeal genomes using *Woltka* with the *Bowtie2* aligner [27], to generate an operational genomic unit (OGU) table and a gene table. The sequence alignments were classified using species taxonomic rank. Samples with insufficient sequencing depth (<100,000 reads after bioinformatic processing) were excluded from downstream analyses. After all quality control steps, the final sequencing depth across retained samples was 797,486 ± 464,956 reads (mean ± SD). Of the 1988 female samples with initial sequencing data, 1526 samples passed all quality control and selection criteria and were eventually included in the present analyses. Details of the pre- and post-sequencing methods and quality control are described in the ESM Methods.

### Serum metabolomics measurement

In the HCHS/SOL, serum metabolomic profiling was performed on 6180 participants at baseline and on 814 participants at visit 2 using an untargeted liquid chromatography-tandem mass spectrometry (LC-MS/MS)-based metabolomic quantification protocol (DiscoveryHD4 platform, Metabolon, Durham, NC, USA). The serum metabolomic profiling at visit 2 was conducted among those who provided blood samples within 1 month of stool collection. Across all detected metabolites, the representative relative SD was 10%. We included 646 named metabolites identified at both visits with coefficients of variation ≤20% among quality control samples and a detection rate >80%. Missing values were replaced by half the minimum detected value for each metabolite. Metabolite concentrations were rank-based inverse-normal transformed prior to analysis. Details of the experiment methods and sample-, instrument- and data-level quality control are described in the ESM Methods.

### Type 2 diabetes ascertainment

Participants were classified as having type 2 diabetes if they reported using glucose-lowering medications or met any of the following American Diabetes Association diagnostic criteria: (1) fasting plasma glucose ≥7.0 mmol/l; (2) 2 h oral glucose tolerance test plasma glucose ≥11.1 mmol/l; and (3) haemoglobin A_1c_ (HbA_1c_) ≥48 mmol/mol (6.5%).

### Metabolic traits assessment

At visit 1 and visit 2, fasting glucose, 2 h glucose, HbA_1c_, fasting insulin, homeostatic model assessment for insulin resistance (HOMA-IR), homeostasis model assessment for beta cell function (HOMA-β), triglyceride, high-density lipoprotein cholesterol (HDL-C), body mass index (BMI) and waist-to-hip ratio (WHR) were measured. At visit 3, only fasting glucose, HbA_1c_, triglyceride, HDL-C, BMI and WHR were measured.

### Statistical analysis

The analytic strategy consisted of three major steps (Fig. 1). Details on covariates and model adjustments are described in the ESM Methods. All analyses were implemented in R (version 4.3.1).

#### Microbiome data preparation

Microbial species present in fewer than 20% of study samples were excluded. Species-level abundances were centred log-ratio (CLR) transformed prior to multivariable linear regression analyses. Indices of α-diversity (observed species richness, Shannon index, Simpson index and Faith’s phylogenetic diversity) and β-diversity (Jensen–Shannon divergence and generalised UniFrac) were calculated from the OGU table using the ‘phyloseq’, ‘picante’ and ‘GUniFrac’ packages in R [28–30].

We considered two models for multivariable adjustment. Model 1 adjusted for sociodemographic characteristics and microbiome-related variables. Model 2 further adjusted for lifestyle factors, medical history and women’s reproductive factors. For gut microbiome α-diversity, we used multivariable linear regression models to examine the association of GDM history with α-diversity indices. For β-diversity, we used permutational multivariate analysis of variance (PERMANOVA) with 999 permutations and multivariable adjustment to compare the differences in overall microbial community composition between women with and without a history of GDM. For abundance of microbial species, we used the analysis of composition of microbiomes (ANCOM; the ‘ancom’ function in ‘ANCOMBC’, R package) [31] to identify the abundance of species different in women with and without a history of GDM, adjusting for covariates in Model 1. An ANCOM detection level ≥0.7 indicates that the ratios of the species to at least 70% of other species are detected to be different (false discovery rate [FDR]-adjusted *p* value [*q* value] <0.1) between women with and without a history of GDM. This threshold was selected a priori for exploratory, hypothesis-generating analyses in the high-dimensional microbiome setting. Subsequently, we used confirmatory multivariable linear regressions to determine the direction and magnitude of the associations per species, with GDM history as a predictor and CLR-transformed microbial species abundance (standardised by SD) as the outcomes, adjusting for covariates in Model 1 and Model 2. To represent the composite effect of GDM on microbiome profile and relate with other types of data, we constructed a GDM-related microbiome score by standardising CLR-transformed abundances of the GDM-associated species and summing or subtracting them based on their association directions with GDM history. We examined the prospective associations of GDM-related individual species and microbiome score with incident type 2 diabetes between visit 2 and visit 3 using multivariable modified Poisson regression.

We performed partial Spearman correlations between identified GDM-related species and 646 serum metabolites at visit 2, to select the metabolites that were correlated with at least one species (FDR-*q*<0.1; multiple testing was controlled across all identified species and metabolite tests). Multivariable linear regression was further used to identify whether the selected metabolites were also associated with a history of GDM at visit 1 (*p*<0.05), adjusting for covariates specified in Model 1. Among these metabolites, those showing the same direction of associations in ‘history of GDM ~ metabolite at visit 1’ and ‘GDM-related microbiome score ~ metabolite at visit 2’ were selected for subsequent analyses, as they are more likely to play a role in linking the GDM-related microbiome to type 2 diabetes. Then, we applied Weibull accelerated failure time (AFT) models to estimate hazard ratios (95% CI) for the associations between the selected metabolites at visit 1 and incident type 2 diabetes over approximately 12 years of follow-up among women without type 2 diabetes at visit 1, with covariates adjustment in Model 2. Since type 2 diabetes was assessed at visits 2 and 3, incident type 2 diabetes during the follow-up visits was considered as interval-censored time-to-event data. Due to the timing of gut microbiome data at visit 2, direct prospective analysis of the gut microbiome with incident type 2 diabetes was constrained by relatively short follow-up time and limited sample size. Therefore, we conducted proxy association analysis [32] using the selected metabolites as proxy measures for the gut microbiome, to explore the prospective associations of GDM-related species and microbiome score with incident type 2 diabetes. Specifically, we correlated the standardised effect size (i.e. natural log-transformed hazard ratios) for associations between selected metabolites and incident type 2 diabetes with the standardised effect size (i.e. β coefficients) for associations between metabolites and GDM-related species or microbiome score. Spearman’s *ρ* between these two sets of effect estimates indicates the prospective relationship of the GDM-related gut microbiome with type 2 diabetes. We also performed mediation analyses with bootstrapping for the selected metabolites (‘mediation’, R package) [33]. We selected regression-based mediation analysis because our goal was to explore the prespecified indirect effect of metabolite features (i.e. metabolite score and identified individual metabolite), which is suitable for observed variables and the available sample size. Analyses were restricted to the metabolites that were associated with both a history of GDM and type 2 diabetes in the multivariable analyses described above, thereby meeting the requisite conditions for mediation analysis. Similarly, metabolite score was created for each participant, by summing or subtracting the inverse-normal transformed levels of these metabolites based on their association directions with GDM history. We performed partial Spearman correlation analysis to assess the cross-sectional correlations of the selected metabolites and metabolite score with metabolic traits at visit 1 with covariates adjustment specified in Model 2. Furthermore, we assessed the correlations of selected metabolites and the metabolite score with the longitudinal changes in metabolic traits.

Additionally, we performed stratified analyses by Hispanic/Latino background and BMI category for the associations between GDM history and identified microbial and metabolite features, and we tested the interaction effect by introducing a product term (GDM history × stratified variable) in the model. Sensitivity analyses were conducted by restricting to pre-menopausal women and applied a different species filtering threshold (those with a prevalence <10% were excluded).

## Results

### Participant characteristics

Our analysis of GDM history and the gut microbiome included 1525 parous women at HCHS/SOL visit 2. The median age was 58 (IQR 52–64) years, and 168 women (11%) reported a history of GDM before visit 2 (Table 1). Compared with women without a history of GDM, those with a history of GDM were younger and more likely to be pre-menopausal, and there were higher proportions of the latter from the Chicago site and of Mexican heritage. They also had a higher Alternative Healthy Eating Index (AHEI)-2010 score, estimated glomerular filtration rate (GFR), gravidity and prevalence of type 2 diabetes. Analyses of GDM history and microbiome-related metabolites included 2968 parous women at visit 1 (ESM Table 1), with similar differences between women with and without prior GDM.

**Table 1:** Characteristics of parous women with gut microbiome data available in HCHS/SOL visit 2, by history of gestational diabetes (*N*=1525)

Characteristic	Women without a history of GDM	Women with a history of GDM	*p* value^a^
*N* (%)	1357 (89)	168 (11)	
Age, years, median (Q1, Q3)	58 (52, 64)	53 (50, 59)	<0.001
Field centre, *n* (%)			0.010
Bronx	365 (27)	43 (26)	
Chicago	344 (25)	56 (33)	
Miami	286 (21)	19 (11)	
San Diego	362 (27)	50 (30)	
Hispanic/Latino background, *n* (%)			0.004
Dominican	157 (12)	19 (11)	
Central or South American	225 (17)	21 (13)	
Cuban	164 (12)	10 (6)	
Mexican	555 (41)	92 (55)	
Puerto Rican	218 (16)	25 (15)	
Mixed/missing	38 (3)	1 (1)	
Educational attainment, *n* (%)			0.783
Less than high school	564 (42)	72 (43)	
Some high school	288 (21)	30 (18)	
High school graduate/equivalent	201 (15)	27 (16)	
More than high school	304 (22)	39 (23)	
Income, *n* (%)			0.223
Less than $30,000	836 (62)	113 (67)	
$30,000 or more	436 (32)	49 (29)	
Missing	85 (6)	6 (4)	
Cigarette use, *n* (%)			0.156
Never	949 (70)	127 (76)	
Former	270 (20)	23 (14)	
Current	138 (10)	18 (11)	
Alcohol use, *n* (%)			0.372
Never	350 (26)	44 (26)	
Former	342 (25)	50 (30)	
Current	665 (49)	74 (44)	
GPAQ total physical activity^b^, MET-min/day, median (Q1, Q3)	114 (0, 411)	137 (9, 487)	0.127
US born, *n* (%)	145 (11)	15 (9)	0.483
Anti-hypertensive medication use, *n* (%)	470 (35)	65 (39)	0.299
Lipid-lowering medication use, *n* (%)	105 (8)	17 (10)	0.283
Estimated GFR, ml/min per 1.73 m^2^, median (Q1, Q3)	101 (88, 111)	106 (90, 118)	0.001
AHEI-2010^b^, median (Q1, Q3)	63 (53, 72)	67 (56, 74)	0.045
BMI, kg/m^2^, median (Q1, Q3)	30 (27, 34)	30 (26, 35)	0.503
Diabetes status, *n* (%)			<0.001
No diabetes	357 (26)	30 (18)	
Prediabetes	653 (48)	53 (32)	
Diabetes	347 (26)	85 (51)	
Antibiotics use^c^, *n* (%)	407 (30)	55 (33)	0.465
Probiotics use^c^, *n* (%)	131 (10)	12 (7)	0.292
Postmenopausal, *n* (%)	1117 (82)	118 (70)	<0.001
Gravidity, median (Q1, Q3)	3 (2, 5)	4 (3, 5)	0.017

^a^ *p* values comparing women with and without a history of GDM are derived from Wilcoxon rank-sum tests for continuous variables and χ^2^ or Fisher’s exact tests (if the expected count is <5) for categorical variables

^b^ GPAQ total physical activity and AHEI-2010 were retrieved from visit 1

^c^ Self-reported use of antibiotics and probiotics in the past 6 months was collected only for participants who provided stool samples

GPAQ, global physical activity questionnaire; MET, metabolic equivalent; Q, quartile

### History of GDM and gut microbiome features

A history of GDM was not associated with any gut microbiome α-diversity indices (*p*>0.2), after adjusting for sociodemographic and microbiome-related covariates (Model 1; ESM Table 2). The results were similar after further adjusting for lifestyle factors and medication use. A history of GDM was not associated with overall gut microbiome composition, assessed by β-diversity, in PERMANOVA models with covariates adjustment in Model 2 (ESM Fig. 1).

**Fig. 2:**
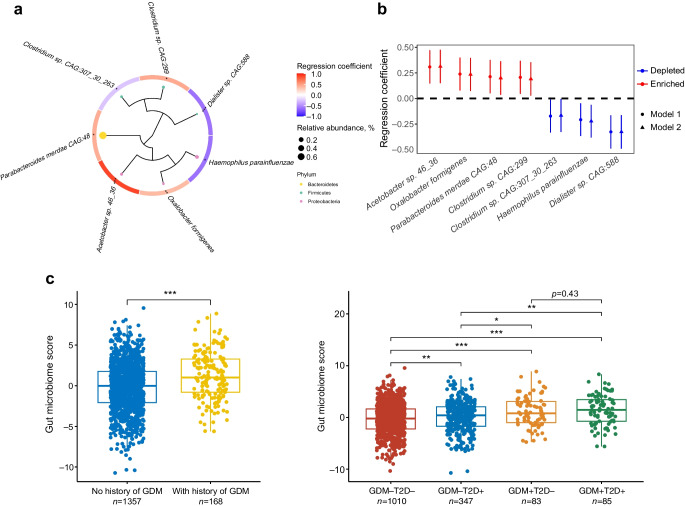
Gut microbiome species abundance and GDM-related microbiome score differed between women with and without a history of GDM (*n*=1525). (**a**) The phylogenetic tree included seven microbial species from three taxonomic phyla that were associated with a history of GDM. The species were identified via ANCOM with covariate adjustment (detection level ≥0.7 and FDR-*q*<0.1), including age, field centre, Hispanic/Latino background, educational attainment, income level, US nativity, antibiotic use and probiotic use. Node colour indicates taxonomic phylum, and node size represents the mean relative abundance. The circular heatmap displays the effect size from confirmatory multivariable linear regression, with GDM history (presence vs absence) as the predictor and CLR-transformed species abundance as the outcome, adjusting for same covariates. (**b**) Multivariable linear regression shows the association of GDM history with CLR-transformed abundance (standardised by the SD) of the seven identified species. Model 1 adjusted for the covariates mentioned in (**a**). Model 2 additionally adjusted for cigarette use, alcohol use, BMI, anti-hypertensive medication use, lipid-lowering medication use, physical activity, AHEI-2010, menopause status and gravidity. Among these, only physical activity and AHEI-2010 were retrieved from visit 1, and all other covariates were collected or measured at visit 2. Regression coefficients represent the difference in standardised CLR-transformed abundance in women with a history of GDM compared with those without. ‘Enriched’ indicates that the microbial species had a higher abundance in women with a history of GDM compared with those without; conversely, ‘depleted’ indicates a lower abundance in women with a history of GDM than in those without. (**c**) GDM-related gut microbiome score differed by GDM history and type 2 diabetes status. Data in (**c**) are presented as box and whisker plot showing the median (centre line), first quartile (Q1), and third quartile (Q3) (box). The whiskers extending from the box show variability outside the upper and lower quartiles. Individual data points are overlaid; *p<0.05, **p<0.01, ***p<0.001. T2D, type 2 diabetes

Of the 1173 microbial species examined, seven species from three taxonomic phyla differed between women with and without a history of GDM (ANCOM detection level ≥0.7 and FDR-*q*<0.1; Fig. 2a, ESM Table 3). *Acetobacter sp. 46_36*, *Parabacteroides merdae CAG:48*, *Clostridium sp. CAG:299* and *Oxalobacter formigenes* had higher abundances in women with a history of GDM than in those without, while *Clostridium sp. CAG:307_30_263*, *Dialister sp. CAG:588* and *Haemophilus parainfluenzae* had lower abundances in women with a history of GDM. The results were consistent in subsequent confirmatory multivariable linear regression (Fig. 2b). These seven species showed weak correlations with each other (Spearman’s *ρ* ranged from −0.19 to 0.37), with similar correlation patterns in women with and without a history of GDM (ESM Fig. 2). In the stratified analysis by prevalent type 2 diabetes, the direction of associations between GDM history and species remained consistent, although the smaller sample size in the subgroups resulted in wider CIs (ESM Table 4). The association of GDM history with the seven species was similar for women with and without prevalent type 2 diabetes (correlation of β coefficients: 0.79, *p*<0.05; ESM Fig. 3b).

The gut microbiome score derived from the seven GDM-related species was higher in women with a history of GDM than in those without (*p*<0.001; Fig. 2c). When categorising jointly by GDM history and type 2 diabetes status, additional differences were observed: the score was higher in women with a history of GDM than in those without, regardless of type 2 diabetes status (*p*<0.001 among women without type 2 diabetes and *p*<0.01 among women with type 2 diabetes); among women without a history of GDM, the score was higher in women with type 2 diabetes than in those without (*p*<0.01); among women with a history of GDM, the score did not differ by type 2 diabetes status, with very limited sample size in these two subgroups (*n*=83 and *n*=85).

### GDM-related gut microbiome and type 2 diabetes

In the cross-sectional analysis, *Clostridium sp. CAG:307_30_263*, depleted in women with a history of GDM, was inversely associated with higher odds of having type 2 diabetes (*p*<0.001) and *O. formigenes*, enriched in women with a history of GDM, was associated with higher odds of having type 2 diabetes (*p*<0.05). A higher GDM microbiome score was associated with a higher prevalence of type 2 diabetes (*p*<0.001; ESM Table 5). Among the 925 women without type 2 diabetes at visit 2, 104 (11.2%) developed type 2 diabetes over 6 years of follow-up. GDM history at visit 2 was associated with a higher incident type 2 diabetes risk, but did not reach statistical significance (Model 1, RR 1.72, *p*=0.053; Model 2, RR 1.46, *p*=0.162; ESM Fig. 4), suggesting that the small sample size and short follow-up limited the ability to detect this well-known association. No association was observed between GDM-related species and incident type 2 diabetes (*p*>0.05). Nevertheless, species associated with GDM history generally demonstrated consistent directional associations with type 2 diabetes.

### GDM-related microbial species, metabolites and type 2 diabetes

**Fig. 3:**
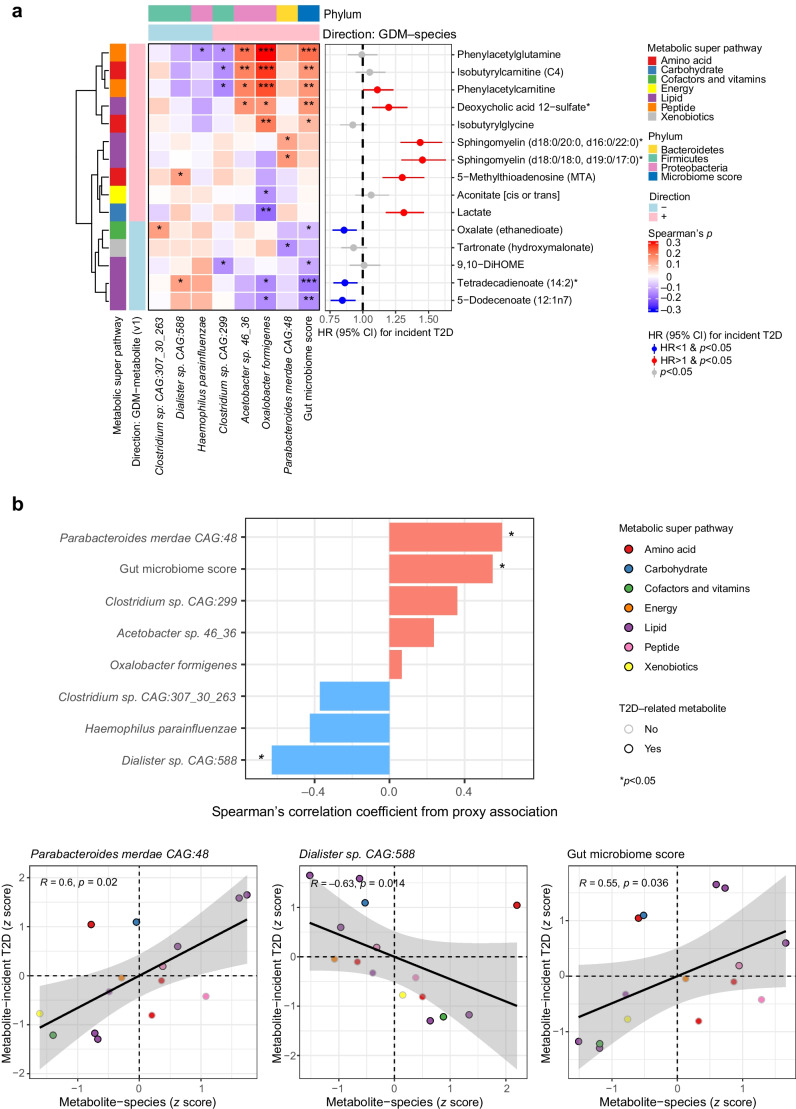
Association between GDM-related gut microbiota, serum metabolites and risk of T2D. (**a**) The heatmap presents partial Spearman correlations of seven microbial species and the GDM-related microbiome score with metabolites, adjusting for age, field centre and estimated GFR (*n*=391, visit 2). The row labelled ‘Direction: GDM–metabolite (v1)’ annotates the direction of association between a history of GDM and inverse-normal transformed metabolite levels at visit 1 (*n*=2968), estimated using multivariable linear regression with adjustment for age, field centre, Hispanic/Latino background, educational attainment, income level and US nativity. ‘*’ denotes metabolites with tentative structural identification (e.g. unresolved isomers) based on Metabolon annotation. The forest plot displays hazard ratios and 95% CIs for the association between each metabolite (measured at visit 1) and incident type 2 diabetes between visit 1 and visit 3 (*n*=2341 women free of prevalent type 2 diabetes at visit 1 and 404 incident cases occurred during the follow-up of approximately 12 years), using Weibull AFT models for interval-censored time-to-event data. The models adjusted for the covariates listed above plus smoking, alcohol use, BMI, anti-hypertensive medication use, lipid-lowering medication use, physical activity, AHEI-2010, estimated GFR, menopause status and gravidity. (**b**) The proxy association is shown between GDM-related microbial species, microbiome score and type 2 diabetes, using 15 metabolites as proxy measures. Scatterplots are shown to visualise the correlations for which Spearman’s *ρ* had *p*<0.05. T2D, type 2 diabetes

A total of 158 out of 646 serum metabolites were correlated with at least one GDM-related species (*n*=391 at visit 2, FDR-*q*<0.1; ESM Table 6), and 35 of them were also associated with a history of GDM at visit 1 (*n*=2968, *p*<0.05; ESM Table 7). Of these 35 metabolites, 15 showed consistent directions in both their associations with GDM history and their correlations with GDM microbiome score (ESM Table 7, ESM Fig. 5). Among 2341 women without prevalent type 2 diabetes at visit 1, 345 (14.7%) developed type 2 diabetes during ~12 years of follow-up. Multivariable Weibull AFT models showed that 9 of these 15 metabolites were prospectively associated with incident type 2 diabetes, including higher levels of six metabolites (e.g. amino acid derivatives and saturated sphingomyelins [SMs]) and lower levels of three metabolites (e.g. fatty acids) (all *p*<0.05; Fig. 3a, ESM Table 7). These nine metabolites showed the same direction of association with GDM history and incident type 2 diabetes. Additionally, GDM microbiome score was positively correlated with deoxycholic acid 12-sulfate* and phenylacetylcarnitine, which were associated with a higher type 2 diabetes risk, while inversely correlated with oxalate, tetradecadienoate (14:2)* and 5-dodecenoate (12:1n7), which were associated with a lower type 2 diabetes risk. To validate the observed correlations between species and metabolites, we performed look-ups for our species–metabolite correlations using the published summary statistics from the Swedish CArdioPulmonary bioImage Study [34] (*n*=8583) and found consistent positive correlations between *P. merdae* and SM (d18:0/18:0, d19:0/17:0)* (*r*=0.02, *p*<0.05) and between *O. formigenes* and isobutyrylglycine (*r*=0.04, *p*<0.001), phenylacetylglutamine (*r*=0.07, *p*<0.001) and phenylacetylcarnitine (*r*=0.05, *p*<0.001).

The proxy association analysis, using the 15 metabolites as proxy measures, suggested that GDM microbiome score (*r*=0.55, *p*=0.036) and *P. merdae CAG:48* (*r*=0.6, *p*=0.02) were positively associated with incident type 2 diabetes, while *Dialister sp. CAG:588* was inversely associated with incident type 2 diabetes (*r*=–0.63, *p*=0.014) (Fig. 3b).

**Fig. 4:**
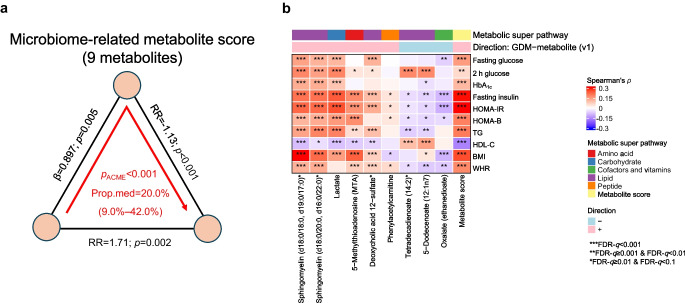
Gut microbiome-related metabolites in GDM-related type 2 diabetes risk. (**a**) The figure shows the mediating role of selected metabolites in the association between a history of GDM and incident type 2 diabetes. Here, we considered the metabolite score consisting of nine metabolites as the potential mediator. These nine metabolites were selected because they were associated with both GDM history and type 2 diabetes in a consistent direction. *p*_ACME_ indicates the *p* value for the indirect effect and Prop.med denotes the proportion of indirect effect divided by the total effect, which were estimated by mediation analyses with adjustment for age, field centre, Hispanic/Latino background, educational attainment, income level and US nativity. (**b**) Partial Spearman correlation of nine serum metabolites and metabolite score with metabolic traits (visit 1, *n*=2580, except for *n*=2393 available for 2 h blood glucose after oral glucose tolerance test), adjusting for age, field centre, Hispanic/Latino background, educational attainment, income level, US nativity, smoking, drinking, BMI (not for the analysis for BMI trait), anti-hypertensive medication use, lipid-lowering medication use, physical activity, AHEI-2010, estimated GFR, menopause status and gravidity. Women with glucose-lowering medication use at visit 1 were excluded. TG, triglyceride

Mediation analysis indicated that 20.0% (95% CI 9.0%, 42.0%) of the association between GDM history and incident type 2 diabetes might be mediated through the metabolite score based on the nine microbiome-related metabolites (*p* value of the average causal mediation effect [*p*_ACME_] <0.001; Fig. 4a). For individual metabolites within the metabolite score, SM (d18:0/18:0, d19:0/17:0)*, SM (d18:0/20:0, d16:0/22:0)* and 5-dodecenoate (12:1n7) may mediate 11.1% (*p*_ACME_=0.06), 12.8% (*p*_ACME_=0.02) and 3.6% (*p*_ACME_=0.04), respectively, of the association between a history of GDM and incident type 2 diabetes (ESM Table 8).

### Gut microbiome-related metabolites and metabolic traits

In the cross-sectional analysis between metabolite features and metabolic traits at visit 1, the microbiome-related metabolite score and six individual metabolites, which were higher in women with a history of GDM, were generally correlated with a worse metabolic profile. By contrast, three metabolites, which were lower in women with a history of GDM, were generally correlated with a more favourable metabolic profile (Fig. 4b). The metabolite score was correlated with increases over time in fasting glucose and HbA_1c_ and decreases over time in WHR during both ~6 and ~12 years of follow-up (ESM Fig. 6a, b). Notably, metabolite score and most metabolites were correlated with increases over time in 2 h blood glucose over ~6 years of follow-up. ESM Fig. 7 summarises all of the identified microbial species and serum metabolites at each step, to provide a clear overview of the analytical workflow.

### Stratified and sensitivity analyses for identified species and metabolites

In stratified analysis by Hispanic/Latino background, no effect modification was observed for GDM history or gut microbiome features, except for *Clostridium sp. CAG:299* (*p*-interaction=0.035), with a stronger association among women of Cuban background (ESM Table 9). No effect modification by Hispanic/Latino background was observed for GDM history and serum metabolite features (all *p*-interaction >0.10; ESM Table 9). In stratified analyses by BMI category, associations between a history of GDM and microbiome and metabolite features were generally directionally consistent across strata, with no effect modification by BMI (all *p*-interaction >0.10; ESM Table 10). In sensitivity analyses using a 10% prevalence threshold, nine microbial species were associated with a history of GDM (ESM Table 11), seven of which overlapped with those identified in the primary analysis. In another sensitivity analysis restricted to pre-menopausal women, associations between a history of GDM and microbial and metabolite features, as well as the mediation effect of the metabolite score on the association between GDM history and incident type 2 diabetes, were generally consistent with those observed in the full cohort (ESM Table 12, ESM Fig. 8).

## Discussion

In this study of US Hispanic/Latino women, we observed higher abundances of four species (e.g. *Parabacteroides* and *Clostridium* species, potentially proinflammatory and pathogenic bacteria) and lower abundances of three species (e.g. *Dialister* species, an SCFA producer with an anti-inflammatory property) in women with a history of GDM than in those without. Integrating with serum metabolomics, we found that nine microbiota-related metabolites (e.g. saturated SMs and unsaturated fatty acids) were associated with various glycaemic traits and incident type 2 diabetes risk. Further proxy and mediation analysis suggested that alterations in GDM-related gut microbiota and serum metabolites may contribute to the relationship between GDM and type 2 diabetes. Our findings suggest that GDM may leave a durable microbial and metabolic imprint that promotes inflammation-mediated insulin resistance, thereby accelerating progression towards type 2 diabetes.

Mounting evidence has shown that there are distinct maternal gut microbiota before or after the diagnosis of GDM, compared with pregnant women without GDM [6–10]. Several recent studies reported that disruptions to the gut microbiome persisted after delivery when GDM was resolved, for up to 16 months postpartum [10, 13–16]. These sustained microbial changes appear to differ from the transient changes observed with GDM during pregnancy and may represent a unique postpartum microbial signature in women with GDM [15]. Notably, the altered gut microbiota profile resembles the dysbiosis patterns observed in non-pregnant women with type 2 diabetes and related intermediary metabolic traits, highlighting their potential relevance in the progression from GDM to type 2 diabetes [14, 15, 17]. However, two other studies did not observe difference in taxonomic abundances between women with and without GDM at 2–6 months [35] and 5 years postpartum [36], which might be due to limited sample size (*n*=84 and *n*=128, respectively). In our study of middle-aged Hispanic/Latino women, we identified seven species that were associated with GDM history. Certain taxa, at the genus level, have been reported in studies conducted during pregnancy [12, 37, 38] and the postpartum period [15], with directions of association with GDM that were consistent with our study. For example, *Parabacteroides* is considered to be a proinflammatory taxon [11] and was reported as the predominant genus contributing to gut microbiome differences between women with and without GDM during mid-pregnancy [12]. *Dialister*, an SCFA-producing genus known to reduce proinflammatory cytokines levels [39], has been found in lower abundance among women with GDM during pregnancy [37, 38], as well as among individuals with type 2 diabetes, compared with those without [40]. Furthermore, different *Clostridium* species may play divergent roles in glycaemic control [12, 15]. These discrepancies may partly reflect the historically poor taxonomic coherence of *Clostridium*, 15 clades within which have been reclassified as the genera *Enterocloster* and *Lacrimispora* in recent years [41]. *Haemophilus* has been reported to be depleted in women with GDM compared with those without during pregnancy [12, 37], as well as in people with diabetes [40]. The reduced abundance of *Haemophilus* may reflect broader gut dysbiosis associated with metabolic dysfunction [42].

Metabolic disturbances from the gut microbiota may contribute to the risk of developing type 2 diabetes in women with prior GDM. We found that GDM-related species were correlated with nine metabolites, and five of them were lipids, including one secondary bile acid and two saturated sphingolipids that were higher in women with a GDM history, while two unsaturated fatty acids were lower in women with a GDM history. Specifically, *P. merdae CAG:48* was positively correlated with two saturated SMs, both of which were associated with an increased risk of type 2 diabetes. *P. merdae CAG:48* is known to produce sphingolipids [43] and may influence circulating sphingolipid levels, which have been implicated in inflammation regulation [44], insulin resistance [45] and type 2 diabetes development [45]. Furthermore, these two SMs showed relatively strong positive correlations with BMI in our study, which is a well-established risk factor for both GDM and type 2 diabetes [46, 47]. *Dialister sp. CAG:588* was positively correlated with tetradecadienoate (14:2)* (a long-chain polyunsaturated fatty acid) and 5-methylthioadenosine (an amino acid derivative that could regulate insulin sensitivity with an anti-obesity property [48]). *Dialister* is an SCFA-producing genus; however, since SCFAs were not measured in either faecal or blood samples in our study, the mechanisms linking *Dialister* to the progression from GDM to type 2 diabetes remain unclear. Moreover, certain species and serum metabolites appear to be uniquely associated with GDM history rather than type 2 diabetes, suggesting the need for further investigation into their biological functions.

Our proxy analysis suggested that the GDM-related microbiome, as reflected by its related metabolites, was prospectively associated with incident type 2 diabetes. Moreover, mediation analysis suggested that certain microbiome-related metabolites may mediate the association between GDM history and type 2 diabetes risk. Most of these metabolites were lipids, aligning with a previous study showing that a lipid-based metabolite score was associated with an increased risk of progression to type 2 diabetes among women with prior GDM [49]. In addition, perturbation of metabolites, potentially shaped by gut microbiota alterations after a pregnancy with GDM, were related to an adverse metabolic profile, particularly characterised by higher levels of glycaemic traits, BMI and WHR. The observed metabolic disturbances may reflect progressive impairments in glucose homeostasis and contribute to an increased risk of type 2 diabetes. Given that obesity is a well-established risk factor for both GDM and type 2 diabetes [46, 47], future studies are needed to further elucidate the interplay of dynamics in adiposity and microbial features in the transition from GDM to type 2 diabetes. Taken together, our results support a unifying mechanistic hypothesis: GDM is associated with durable alterations in the gut microbiome that favour proinflammatory and insulin resistance-promoting metabolic outputs, particularly adverse lipid metabolite, which may lead to impairments in glucose homeostasis and ultimately increase the susceptibility of progression to type 2 diabetes.

This is the first study to investigate potential biological mechanisms underlying the relationship between a history of GDM and subsequent type 2 diabetes using an integrative microbiome–metabolomics approach. However, several limitations should be considered. First, GDM history was self-reported, which may introduce recall bias. Nevertheless, a prior study demonstrated high concordance (~94%) between self-reported and physician-diagnosed GDM obtained from medical records [50]. In our study, GDM history was repeatedly assessed at both visit 1 and visit 2, and additional diagnoses made between visits were documented, which may enhance GDM ascertainment. Second, the timing of GDM diagnosis was not collected, limiting our ability to perform time-to-event analysis. Third, data on relevant lifestyle, metformin use and other pregnancy complications covariates before/at the time of GDM diagnosis were unavailable and may have confounded the observed associations. Fourth, the cross-sectional timing of gut microbiome and serum metabolite measurements (at visit 2) precludes definitive conclusions regarding the causal order and, thus, causal or mediation interpretations should be made with caution. Finally, without validation in other populations and study settings, our findings should be interpreted cautiously with respect to generalisability. Future studies are warranted to longitudinally assess the gut microbiota and metabolites after delivery in well-characterised birth cohorts to validate and expand upon our findings.

### Conclusion

Among US Hispanic/Latino women, a history of GDM is associated with an unfavourable gut microbiota and related serum metabolite profiles, which may contribute to worse glycaemic profiles and a higher type 2 diabetes risk. The observed gut dysbiosis and metabolic perturbations appear to be long-lasting after a pregnancy complicated by GDM, highlighting the potential utility of gut microbiota and circulating metabolites as biomarkers for identifying high-risk women before the onset of overt diabetes. Given that the gut bacteria and intermediate metabolites can be modulated by possible targeted interventions, identifying key microbial players in the transition from GDM to type 2 diabetes can help develop preventive approaches to mitigate type 2 diabetes risks for women with a history of GDM.

## Supplementary Information

Below is the link to the electronic supplementary material.

ESM (PDF 4020 KB)

ESM Tables (XLSX 353 KB)

## Data Availability

The HCHS/SOL data are archived at the National Institutes of Health repositories dbGaP and BIOLINCC. The HCHS/SOL has established a process for the scientific community to apply for access to participant data and materials, with such requests reviewed by the project’s Steering Committee. These policies are described at https://sites.cscc.unc.edu/hchs/. The analysis code supporting the findings of this study has been deposited in Zenodo and is publicly available at 10.5281/zenodo.18445861.

## Abbreviations

AFT: Accelerated failure time
AHEI-2010: Alternative Healthy Eating Index-2010
ANCOM: Analysis of composition of microbiomes
BMI: Body mass index
CLR: Centred log-ratio
FDR: False discovery rate
GDM: Gestational diabetes mellitus
GFR: Glomerular filtration rate
HbA_1c_: Haemoglobin A_1c_
HCHS/SOL: Hispanic Community Health Study/Study of Latinos
HDL-C: High-density lipoprotein cholesterol
OGU: Operational genomic unit
*p*_ACME_: *p* value of the average causal mediation effect
PERMANOVA: Permutational multivariate analysis of variance
SCFA: Short-chain fatty acid
SM: Sphingomyelin
WHR: Waist-to-hip ratio
