# Supplementary material for: Gestational diabetes and risk of type 2 diabetes: exploring the role of the gut microbiome in the Hispanic Community Health Study/Study of Latinos (HCHS/SOL)
Source: Diabetologia. 2026 Apr 30;69(8):2225–39. doi: 10.1007/s00125-026-06727-0 (PMC13310232; doi:10.1007/s00125-026-06727-0)
Supplement: Supplementary file 1 — ESM (PDF 4020 KB) [file 125_2026_6727_MOESM1_ESM.pdf]

ELECTRONIC SUPPLEMENTARY MATERIAL

Gestational Diabetes and Risk of Type 2 Diabetes: Exploring the Role of Gut microbiome in the Hispanic Community Health Study/Study of Latinos (HCHS/SOL)

Yi Wang, Carmen R. Isasi, Alison M. Stuebe, Adetola F. Louis-Jacques, Jie Hu, Gang Hu, Martha L. Daviglus, Eric Boerwinkle, Robert D. Burk, Robert C. Kaplan, Qibin Qi, Brandilyn A. Peters*

***Corresponding author:**
Brandilyn A. Peters, PhD
Associate Professor of Epidemiology
Department of Epidemiology and Population Health
Albert Einstein College of Medicine
1300 Morris Park Avenue, #1315AB
Bronx, NY 10461
718-430-3281 (phone); brandilyn.peterssamuelson@einsteinmed.edu (email)

Electronic Supplementary Material (ESM) Methods

ESM Fig. 1. History of GDM and gut microbiome overall composition (n=1525).

ESM Fig. 2. Spearman correlations among CLR-transformed abundances of seven GDM-related microbial species.

ESM Fig. 3. Association between GDM history and gut microbiota, accounting for T2D status.

ESM Fig. 4. Association of history of GDM and GDM-related gut microbiome with incident type 2 diabetes between visit 2 and visit 3 (n=925).

ESM Fig. 5. Fifteen metabolites exhibit consistent directional associations with GDM-related gut microbiome (visit 2) and history of GDM (visit 1).

ESM Fig. 6. Partial Spearman correlations of 9 serum metabolites and metabolite score with longitudinal changes in clinical metabolic traits between visit 1 and visit 2 (a, n=2580), and visit 1 and visit 3 (b, n=1470).

ESM Fig. 7. Overview of microbial species and serum metabolites selection process.

ESM Fig. 8. Mediating role of selected metabolites in the association between a history of GDM and incident T2D among pre-menopausal women who were free of T2D at visit 1 (n=1314).

ESM Table 1. Characteristics of parous women in the HCHS/SOL visit 1 with serum metabolomics data available by history of gestational diabetes (n=2968).

ESM Table 2. Associations of GDM history with gut microbiome α-diversity (n=1525).

ESM Table 3. Association of GDM history with gut microbial species (n=1525).

ESM Table 4. Association of GDM history with microbial species in women with (n=432) and without (n=1093) prevalent type 2 diabetes at visit 2.

ESM Table 5. Association of GDM-related species and gut microbiome score with prevalent type 2 diabetes (n=1525).

ESM Table 6. Partial Spearman correlations of 7 GDM-related species and microbiome score with 646 named metabolites among a subset of HCHS/SOL women with metabolomics data available at visit 2 (n=391).

ESM Table 7. Associations of 35 selected serum metabolites with incident type 2 diabetes.

ESM Table 8. Mediating role of 9 selected microbiome-related metabolites in the association between history of GDM and incident type 2 diabetes.

ESM Table 9. Association between history of GDM and identified microbial features and metabolite features, stratified by Hispanic/Latino background.

ESM Table 10. Association between history of GDM and identified microbial features and metabolite features, stratified by BMI categories.

ESM Table 11. Microbial species associated with history of GDM after filtering out the species present in <10% of the study samples (n=1525).

ESM Table 12. Association between history of GDM and identified microbial features and metabolite features among pre-menopausal women.

ESM Methods

Study design and sample size

A total of 8780 women had a history of pregnancy prior to visit 2. After excluding women with missing data on GDM history or gut microbiome at visit 2, those with <100K sequence reads in their metagenome sample, and those with prevalent cardiovascular disease or cancer, a total of 1525 women were included in the analysis examining the association between GDM history and gut microbiome. Furthermore, a total of 8765 women reported having been pregnant prior to visit 1. After excluding those with missing data on GDM history or serum metabolomics at visit 1, as well as those with prevalent cardiovascular disease or cancer, a total of 2968 parous women were included in the analysis examining the association between GDM history and serum metabolites.

Our study population included women with existing T2D, as prevalent T2D is more likely to act as a collider (or mediator, but not confounder) in the association between GDM history and the gut microbiome, as illustrated in the directed acyclic graph (**ESM Fig. 3a**), therefore adjusting or restricting based on diabetes status may not be appropriate. Instead, we examined and compared the effect estimates in women with and without prevalent T2D.

First, we identified microbial species differing in women with and without a history of GDM, and then identified serum metabolites correlated with these GDM-related species. Second, we selected the metabolites associated with both GDM history and GDM-related microbiome in the same direction. Third, we examined the prospective associations of the selected metabolites with incident T2D.

Quality control (QC) and normalization for shotgun metagenomics sequencing data

Pre-sequencing: DNA was extracted from stool samples with the MO BIO PowerSoil DNA extraction kit (Carlsbad, CA) following the standardized Earth Microbiome Project protocol [1] (https://www.protocols.io/view/emp-dna-extraction-protocol-4r3l2rkjv1y9/v1). This protocol incorporates rigorous contamination control, standardized mechanical and chemical lysis, sequential inhibitor removal, and membrane-based spin filter purification with ethanol-containing wash steps to ensure high-quality DNA suitable for downstream sequencing. Extracted DNA was quantified in a 384-well plate using a PicoGreen fluorescence assay (ThermoFisher, Inc.) and normalized to 1 ng input DNA using an Echo 550 acoustic liquid-handling robot (Labcyte, Inc.). Library preparation was performed using an automated workflow,

as described previously [2]. Enzyme mixes for fragmentation, end repair and A-tailing, ligation, and PCR were added using a Mosquito HV micropipetting robot (TTP Labtech). Fragmentation was performed at 37°C for 20 min, followed by end-repair and A-tailing at 65°C for 30 min. Sequencing adapters and barcode indices were added in two steps by following the iTru adapter protocol [3]. Universal "stub" adapter molecules and ligase mix were first added to the end-repaired DNA using the Mosquito HV robot and ligation performed at 20°C for 1 h. Unligated adapters and adapter dimers were removed using AMPure XP magnetic beads and a BlueCat purification robot (BlueCat Bio). Next, individual i7 and i5 were added to the adapter-ligated samples using the Echo 550 robot. Eluted bead-washed ligated samples then were added to PCR master mix and PCR amplified for 15 cycles. The amplified and indexed libraries were purified again using magnetic beads and the BlueCat robot, resuspended in water, and transferred to a 384-well plate using the Mosquito HTS liquid-handling robot for library quantitation, sequencing, and storage. Samples were then normalized based on a PicoGreen fluorescence assay for sequencing on Illumina NovaSeq [4].

Post-sequencing: Raw FASTQ sequence reads were processed using the standard shotgun sequencing pipeline implemented in *Qiita* [5]. Per-sample sequence adapters were trimmed via *fastp* [6], and sequence reads mapping to the human reference genome (GRCh38) were identified and removed using *minimap2* (qp-fastp-minimap2 2022.04) [7]. High-quality, non-host reads were then aligned against the WolR1 [8] reference database of bacterial and archaeal genomes using *Woltka* with the *Bowtie2* aligner [9], to generate an operational genomic unit (OGU) table and a gene table. The sequence alignments were classified at species taxonomic rank. Samples with insufficient sequencing depth (<100,000 reads after bioinformatic process) were excluded from downstream analyses. After all QC steps, the final sequencing depth across retained samples was 797,486 ± 464,956 reads (mean ± SD). Of the 1988 female samples with initial sequencing data, 1526 samples passed all QC and selection criteria, and were included in the present analyses.

Normalization and downstream analysis: Microbial species abundance data were centered log-ratio (CLR) transformed prior to regression analyses, and differential abundance testing was performed using ANCOM, which is specifically designed to account for compositional structure of microbiome data and does not require library-size normalization.

QC and normalization for metabolomics data

Sample-level QC: Serum samples were extracted with methanol and analyzed using an established, untargeted ultra-performance liquid chromatography (UPLC)-MS/MS platform [10] (DiscoveryHD4, Metabolon Inc.). Following extraction, samples were dried and reconstituted in method-specific solvents [11]. Each reconstitution solvent contained a series of internal standards at fixed concentrations to ensure injection and chromatographic consistency across all samples. Study samples were randomized across analytical runs to minimize potential batch effects.

Instrument-level QC: Metabolomic profiling was performed using a Waters ACQUITY UPLC and a Thermo Scientific Q-Exactive high resolution/accurate mass spectrometer interfaced with a heated electrospray ionization (HESI-II) source and Orbitrap mass analyzer operated at 35,000 mass resolution. To maximize metabolome coverage, four complementary chromatographic and ionization methods were applied: two reverse phase methods with positive ion mode electrospray ionisation (EI), one reverse phase method with negative ion mode EI, and one hydrophilic interaction liquid chromatography with negative ion mode EI [11, 12]. Instrument performance, stability, and analytical reproducibility were continuously monitored using pooled QC samples generated from study samples and injected repeatedly throughout each analytical run. Instrument variability was assessed by calculating the median relative standard deviation (RSD) of internal standards added to each sample prior to injection into the mass spectrometers. Overall analytical process variability was evaluated by calculating the median RSD of endogenous metabolites (i.e., non-instrument standards) present in 100% of technical replicate samples. The RSD was 10% across all the metabolites detected.

Data-level QC and normalization: Metabolites were identified by automated comparison of the ion features in the experimental samples to a reference library of chemical standard entries that included retention time, molecular weight (m/z), preferred adducts, and in-source fragments as well as associated MS spectra and curated by visual inspection for QC using software developed at Metabolon [13, 14]. Peak areas were quantified using area-under-the-curve. To correct for inter-day instrument variability, raw area counts for each metabolite in each sample were normalized by the median value for each run-day, thereby setting the median to 1.0 for each run. Among the annotated metabolites, those with coefficients of variation (CV)>20% across pooled QC samples or with detection rate <80% were excluded from analysis. Values below the

lower limit of detection were replaced by half the minimum value for each metabolite. Metabolite concentrations were rank-based inverse normal transformed prior to downstream analysis.

Covariate data and model adjustments

Sociodemographic characteristics, lifestyle factors, medical history, women's reproductive factors, and microbiome-related variables (collected only among participants who provided stool samples) were obtained through interviewer-administered questionnaires at each visit, while anthropometric indices were measured via standardized physical examinations. The sociodemographic characteristics used in this study included age (years), field center (Bronx, Chicago, Miami, San Diego), Hispanic/Latino background (Dominican, Central or South American, Cuban, Mexican, Puerto Rican, Mixed/missing), educational attainment (less than high school, some high school, high school graduate/equivalent, more than high school), income (less than \$30,000, \$30,000 or more, missing), and U.S. nativity (born in U.S. 50 states/DC or not); lifestyle factors and medical history included cigarette use (never, former, current), alcohol use (never, former, current), self-reported physical activity (metabolic equivalent [MET]-min/d), Alternative Healthy Eating Index (AHEI)-2010, anti-hypertensive medication use (yes, no), lipid-lowering medication use (yes, no), BMI (kg/m^2), estimated glomerular filtration rate (eGFR, $mL/min/1.73m^2$); women's reproductive factors included menopause status (yes/no) and gravidity (i.e., number of pregnancies); microbiome-related variables included antibiotics use (yes, no) and probiotics use in the past 6 months (yes, no), which were only used in microbiome-related analysis. Among these covariates, physical activity and AHEI-2010 were only assessed at visit 1, whereas others were collected or measured at both visits. Total physical activity was assessed using the Global Physical Activity Questionnaire. Estimated GFR was calculated based on serum creatinine and cystatin C using the new CKD-EPI creatinine-cystatin C equation without race[15]. Covariates from visit 1 or visit 2 were selected based on exposure of interest in different analyses. Missing values in covariates were imputed using the median (for continuous variables) or mode (for categorical variables), with the exception for categorical variables with >1% missing, for which a separate "missing" category was created. Covariates were adjusted using two models: Model 1 adjusted for age, field center, Hispanic/Latino background, educational attainment, income level, U.S. nativity, antibiotics use, and probiotics use; and

Model 2 additionally adjusted for cigarette use, alcohol use, BMI, anti-hypertensive medication use, lipid-lowering medication use, physical activity, AHEI-2010, menopause status, and gravidity.

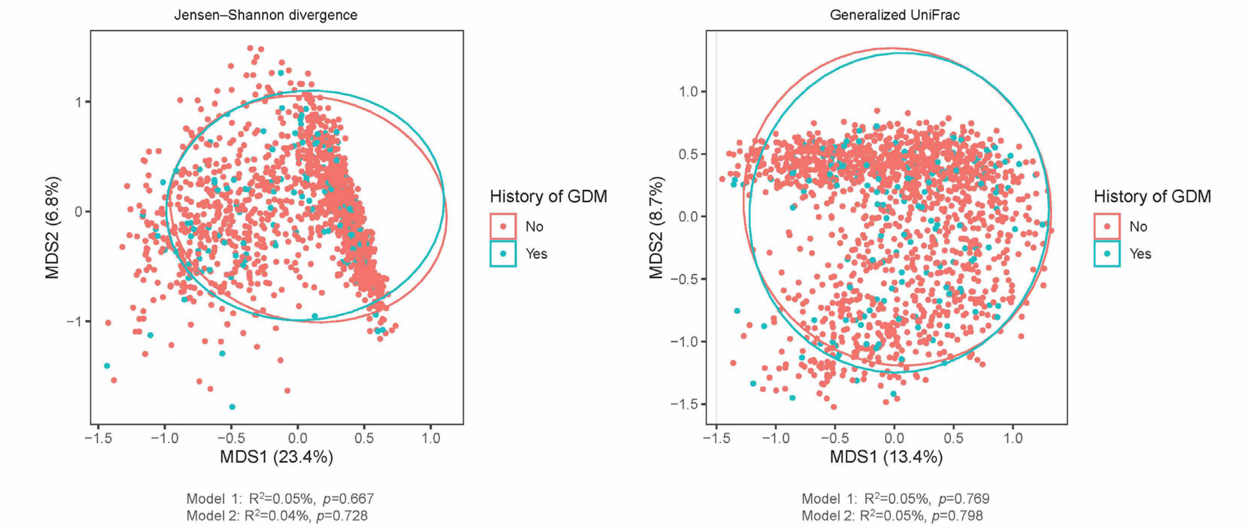

ESM Fig. 1. History of GDM and gut microbiome overall composition (n=1525). Gut microbiome β-diversity indices were assessed by the Jensen-Shannon Divergence (left panel) and generalized UniFrac distance (right panel) at microbial genome level. R-squared and p-values were derived from permutational multivariate analysis of variance, with GDM history as predictor and β-diversity indices as outcomes. Model 1 adjusted for age, field center, Hispanic/Latino background, educational attainment, income level, U.S. nativity, antibiotics use, and probiotics use. Model 2 additionally adjusted for cigarette use, alcohol use, BMI, anti-hypertensive medication use, lipid-lowering medication use, physical activity, AHEI-2010, menopause status, and gravidity. Among these covariates, only physical activity and AHEI-2010 were retrieved from visit 1, and all other covariates were collected or measured at visit 2. AHEI, Alternative Healthy Eating Index; BMI, body mass index; GDM, gestational diabetes mellitus; GFR, glomerular filtration rate.

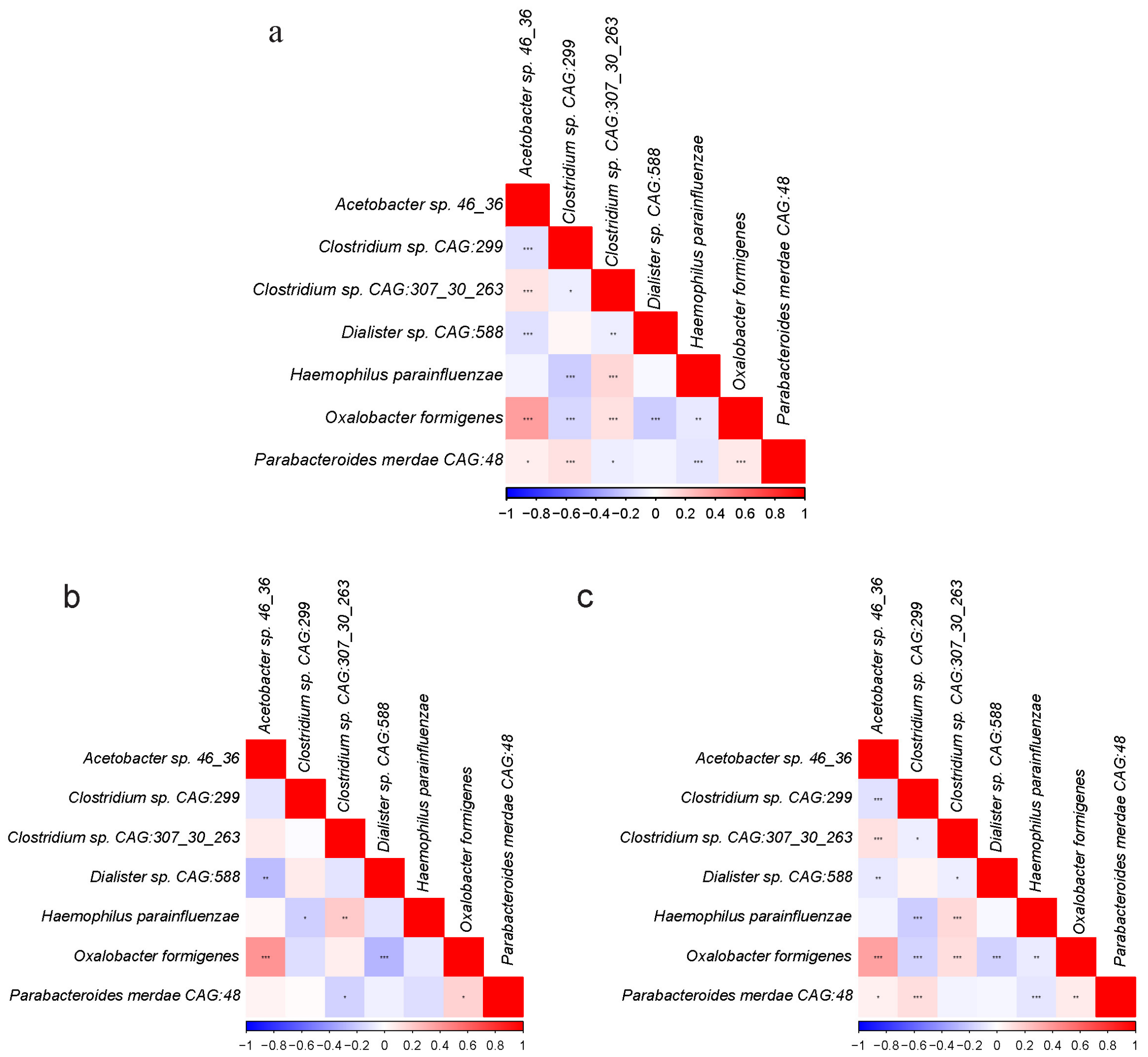

ESM Fig. 2. Spearman correlations among CLR-transformed abundances of seven GDM-related microbial species. Heatmaps of Spearman correlation matrices are shown for (a) all eligible women at Visit 2 (n=1525), (b) women with a history of GDM (n=168), and (c) women without a history of GDM (n=1357). **p*<0.05; ***p*<0.01; ****p*<0.001. GDM, gestational diabetes mellitus.

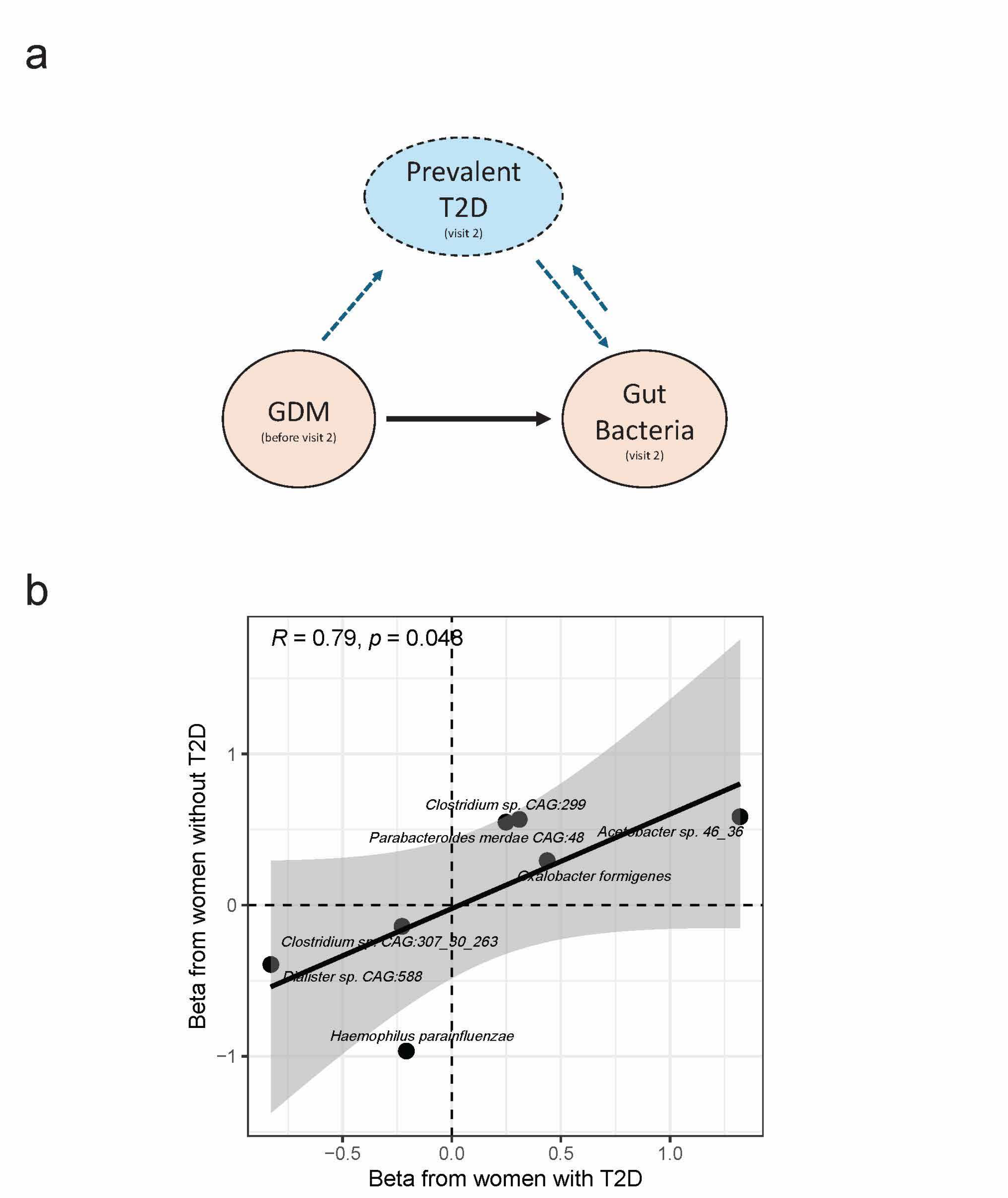

ESM Fig. 3. Association between GDM history and gut microbiota, accounting for T2D status. a. Directed acyclic graph (DAG) shows the role of prevalent T2D in the association between GDM history and gut bacteria. **b**. Correlation of regression coefficients for the association between GDM history and seven microbial species among women with (n=432) and without (n=1093) prevalent T2D at visit 2. Multivariable linear regressions were used to derive the regression coefficients, with GDM history (yes vs. no) as predictor and CLR-transformed abundance of each microbial species as outcome, among women with and without prevalent type 2 diabetes. Models were adjusted for age, field center, Hispanic/Latino background, educational attainment, income level, U.S. nativity, antibiotic use, and probiotic use. CLR, centered log-ratio; GDM, gestational diabetes mellitus; T2D, type 2 diabetes.

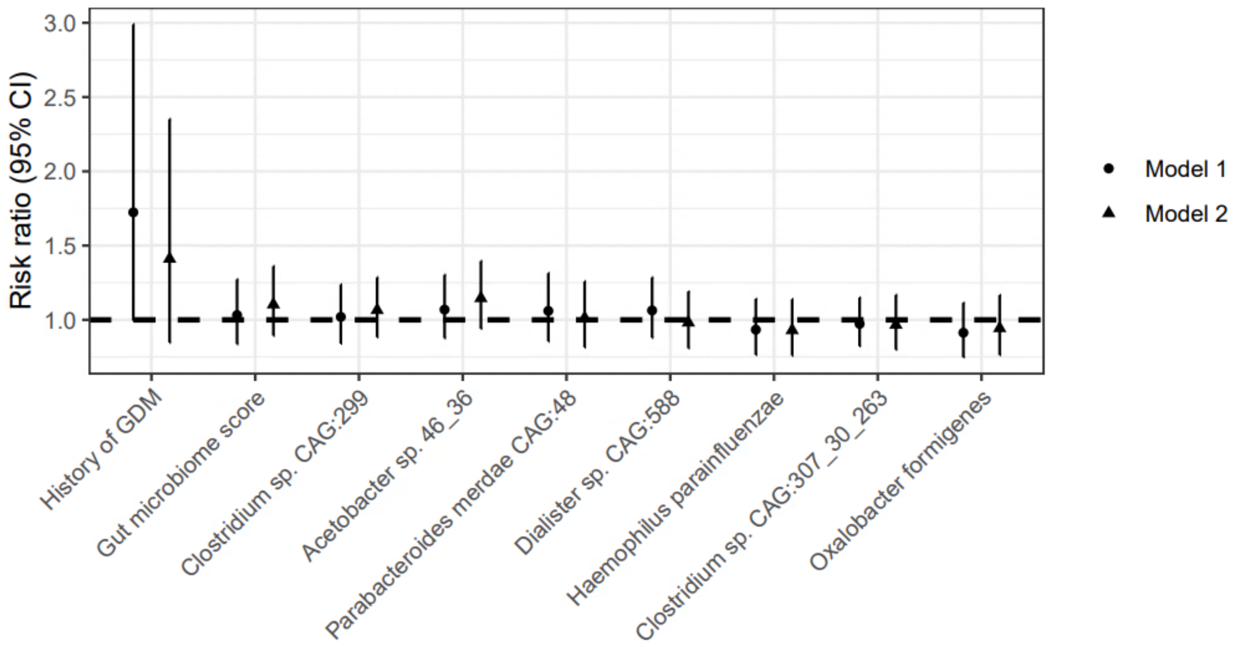

ESM Fig. 4. Association of history of GDM and GDM-related gut microbiome with incident type 2 diabetes between visit 2 and visit 3 (n=925). This analysis excluded women with prevalent type 2 diabetes at visit 2. Multivariable Poisson regression models with a sandwich variance estimator were used to estimate relative risk (95% CI) for the association of each species and microbiome score with incident type 2 diabetes. Relative risk reflects the effect per 1-SD increase in CLR-transformed species abundance or microbiome score. Model 1 adjusted for age, field center, Hispanic/Latino background, educational attainment, income level, U.S. nativity, antibiotics use, and probiotics use. Model 2 additionally adjusted for cigarette use, alcohol use, BMI, anti-hypertensive medication use, lipid-lowering medication use, physical activity, AHEI-2010, menopause status, and gravidity. Among these, only physical activity and AHEI-2010 were retrieved from visit 1, and all other covariates were collected or measured at visit 2. AHEI, Alternative Healthy Eating Index; BMI, body mass index; CLR, centered log-ratio; CI, confidence interval; GDM, gestational diabetes mellitus.

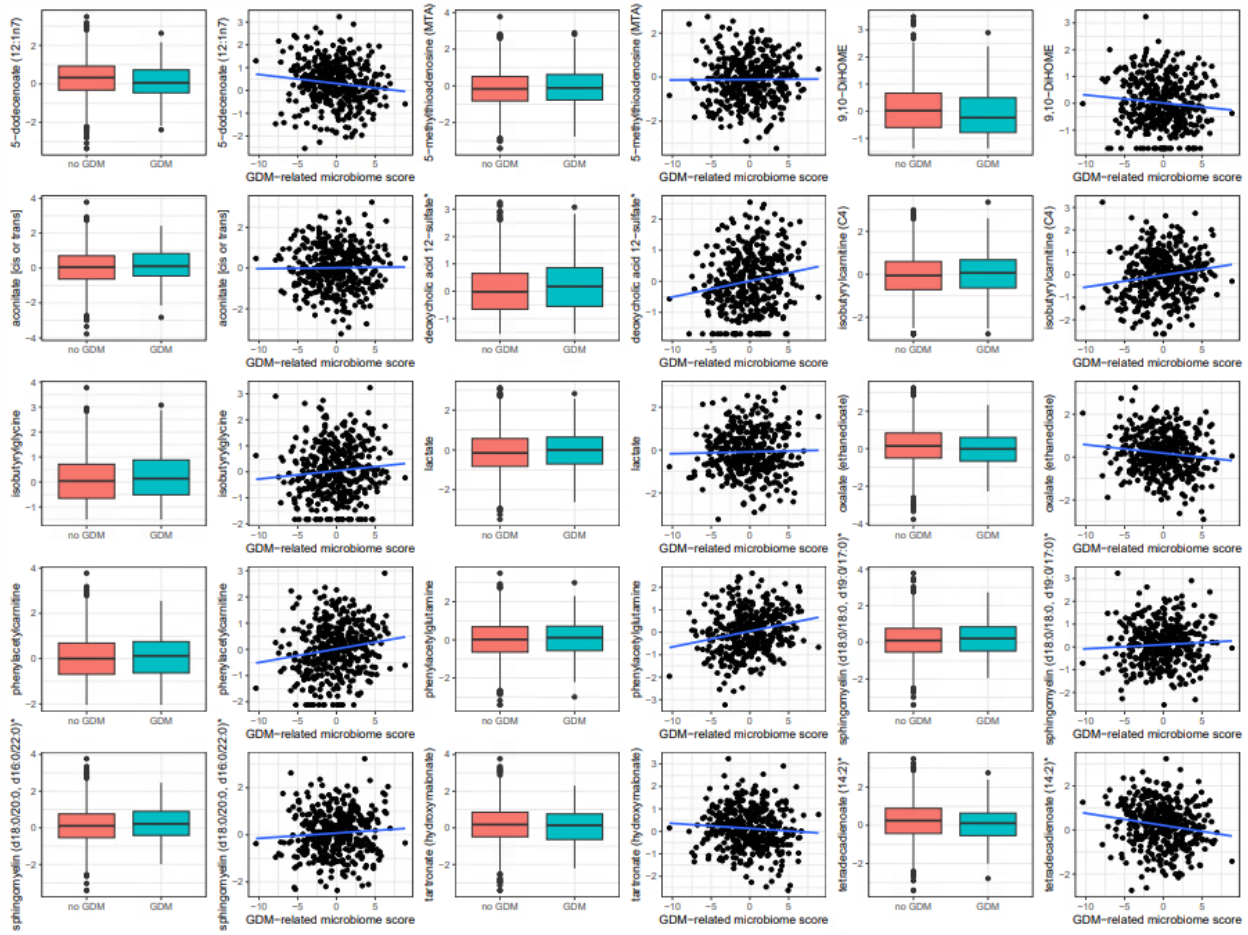

ESM Fig. 5. Fifteen metabolites exhibit consistent directional associations with GDM-related gut microbiome (visit 2) and history of GDM (visit 1). The boxplots show the distributions of inverse-normal transformed serum metabolite levels at visit 1 (n = 2968, 248 women with a history of GDM) among women with and without a history of GDM. The adjacent scatterplots show the correlation between the GDM-related gut microbiome score and serum metabolite levels at visit 2 (n=391). GDM, gestational diabetes mellitus.

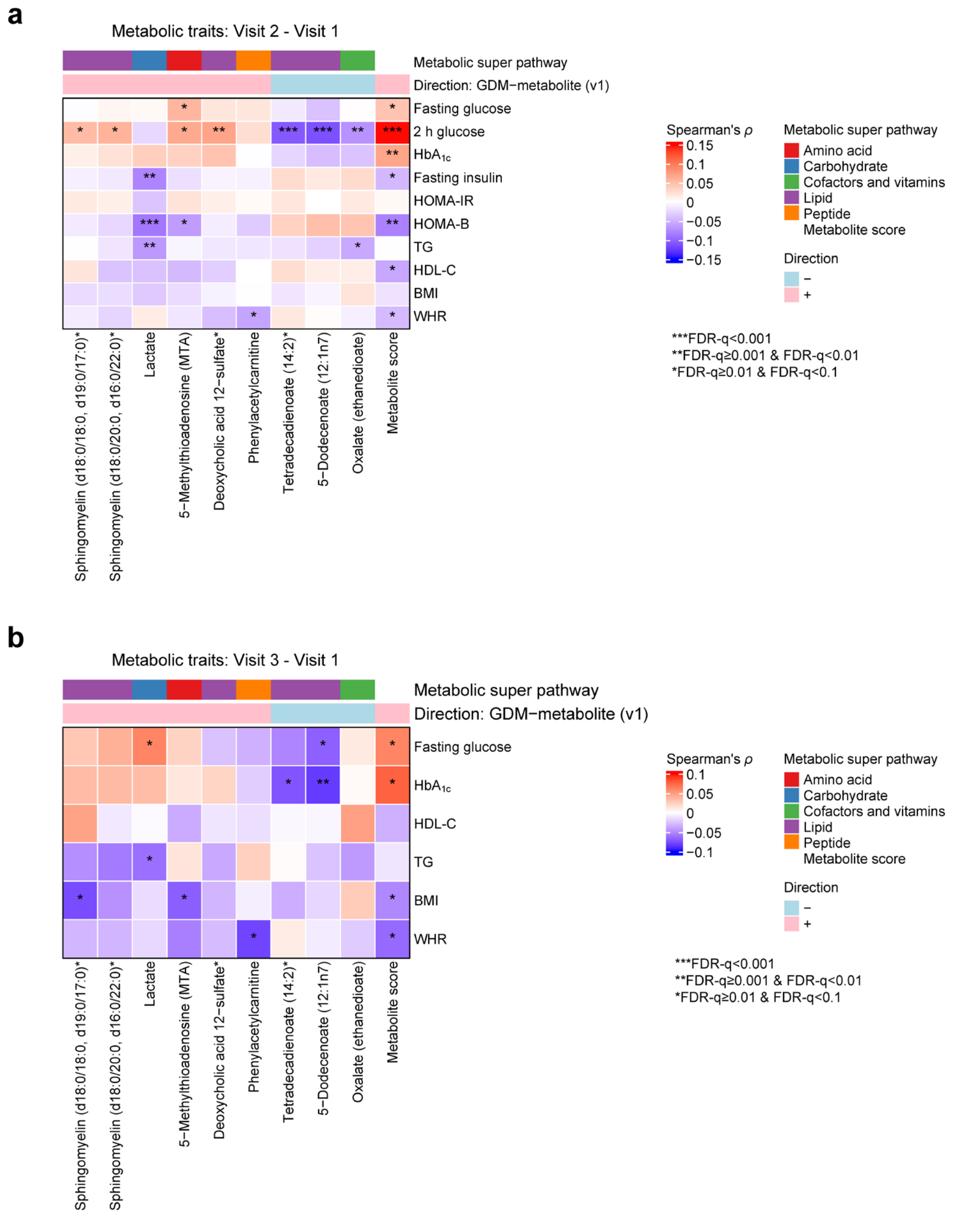

ESM Fig. 6. Partial Spearman correlations of 9 serum metabolites and metabolite score with longitudinal changes in clinical metabolic traits between visit 1 and visit 2 (a, n=2580), and visit 1 and visit 3 (b, n=1470). Partial Spearman correlations adjusted for age, field center, Hispanic/Latino background, educational attainment, income level, U.S. nativity, smoking, drinking, BMI (not for BMI analysis), anti-hypertensive medication use, lipid-lowering medication use, physical activity, AHEI-2010, estimated GFR, menopause status, and gravidity. Women with anti-diabetic medication use at visit 1 were excluded. 2-h glucose, 2-hour blood glucose after oral glucose tolerance test; AHEI, Alternative Healthy Eating Index; BMI, body mass index; GFR, glomerular filtration rate; HDL-C, high-density lipoprotein-cholesterol; HbA_{1c}, hemoglobin A_{1c}; HOMA-β, homeostasis model assessment for β-cell function; HOMA-IR, homeostatic model assessment for insulin resistance; TG, triglyceride; WHR, waist-to-hip ratio.

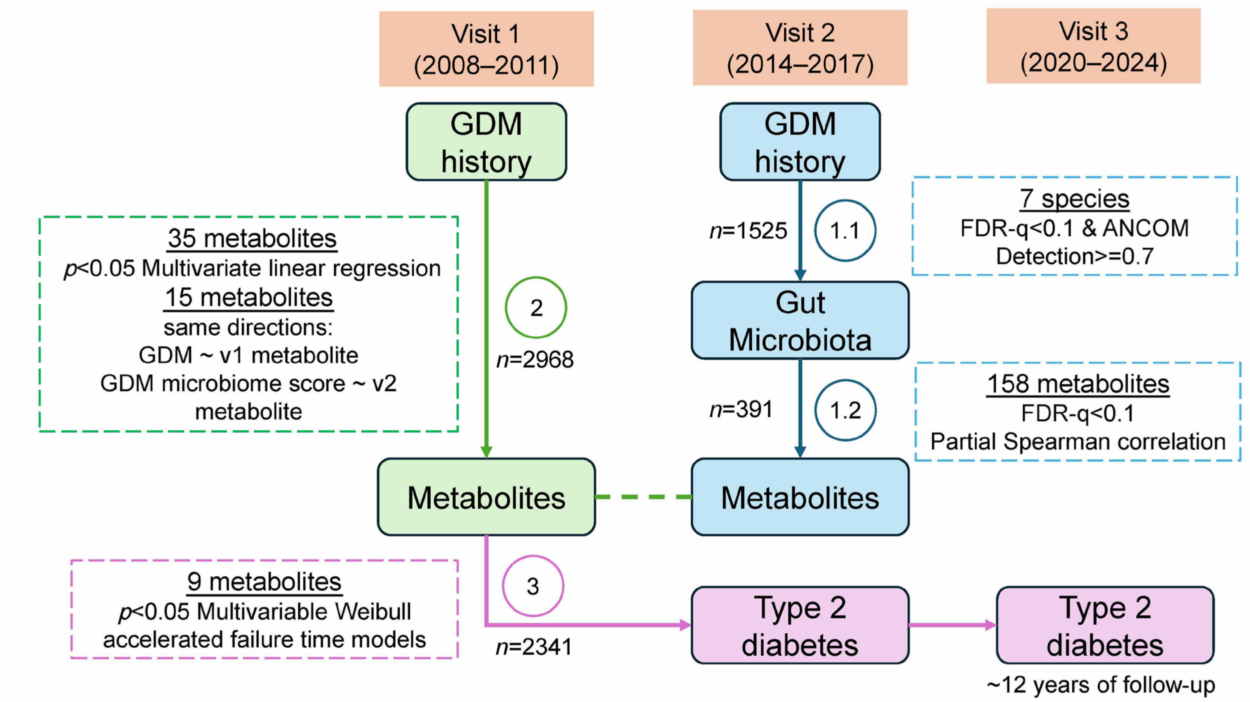

ESM Fig. 7. Overview of microbial species and serum metabolites selection process. This figure illustrates the multi-step analytical framework used to identify the microbial species and serum metabolites linking in GDM-T2D association. Color-coded boxes and directional arrows represent the three major steps of the analysis pipeline. Major statistical approaches are annotated alongside each step, along with the corresponding selected microbial species and serum metabolites at each step. GDM, gestational diabetes mellitus.

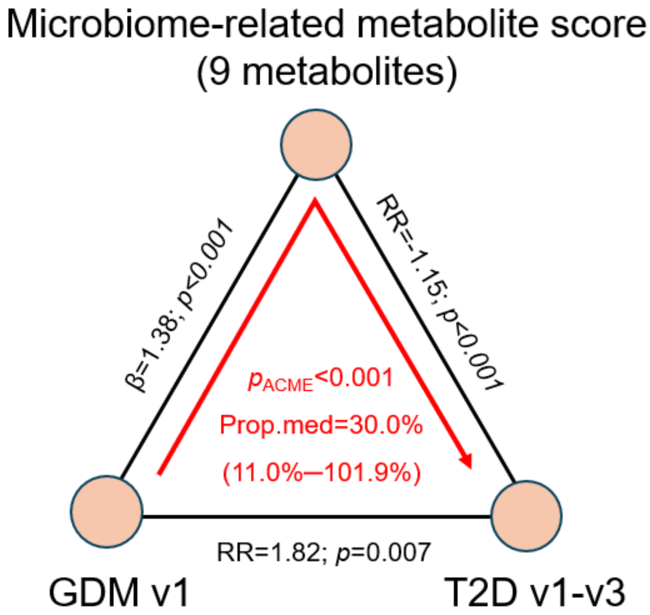

ESM Fig. 8. Mediating role of selected metabolites in the association between a history of GDM and incident T2D among pre-menopausal women who were free of T2D at visit 1 (n=1314). These 9 metabolites were selected because they were associated with both GDM history and T2D in consistent direction. p_{ACME} indicates the p-value for indirect effect and *Prop.med* denotes the proportion of indirect effect divided by the total effect, which were estimated by mediation analyses with adjustment for age, field center, Hispanic/Latino background, educational attainment, income level, and U.S. nativity. ACME, average causal mediation effect; GDM, gestational diabetes mellitus; Prop.med, proportion mediated; T2D, type 2 diabetes.

References:

1. Thompson LR, Sanders JG, McDonald D et al (2017) A communal catalogue reveals Earth's multiscale microbial diversity. Nature 551(7681):457-463. https://doi.org/10.1038/nature24621
2. Peters BA, Lin J, Qi Q et al (2022) Menopause Is Associated with an Altered Gut Microbiome and Estrobolome, with Implications for Adverse Cardiometabolic Risk in the Hispanic Community Health Study/Study of Latinos. mSystems 7(3):e0027322. https://doi.org/10.1128/msystems.00273-22
3. Glenn TC, Nilsen RA, Kieran TJ et al (2019) Adapterama I: universal stubs and primers for 384 unique dual-indexed or 147,456 combinatorially-indexed Illumina libraries (iTru & iNext). PeerJ 7:e7755. https://doi.org/10.7717/peerj.7755
4. Costello M, Fleharty M, Abreu J et al (2018) Characterization and remediation of sample index swaps by non-redundant dual indexing on massively parallel sequencing platforms. BMC Genomics 19(1):332. https://doi.org/10.1186/s12864-018-4703-0
5. Gonzalez A, Navas-Molina JA, Kosciolek T et al (2018) Qiita: rapid, web-enabled microbiome meta-analysis. Nat Methods 15(10):796-798. https://doi.org/10.1038/s41592-018-0141-9
6. Chen S, Zhou Y, Chen Y, Gu J (2018) fastp: an ultra-fast all-in-one FASTQ preprocessor. Bioinformatics 34(17):i884-i890. https://doi.org/10.1093/bioinformatics/bty560
7. Li H (2018) Minimap2: pairwise alignment for nucleotide sequences. Bioinformatics 34(18):3094-3100. https://doi.org/10.1093/bioinformatics/bty191
8. Zhu Q, Mai U, Pfeiffer W et al (2019) Phylogenomics of 10,575 genomes reveals evolutionary proximity between domains Bacteria and Archaea. Nat Commun 10(1):5477. https://doi.org/10.1038/s41467-019-13443-4
9. Langmead B, Salzberg SL (2012) Fast gapped-read alignment with Bowtie 2. Nat Methods 9(4):357-359. https://doi.org/10.1038/nmeth.1923
10. Evans AM, Br B, Liu Q et al (2014) High Resolution Mass Spectrometry Improves Data Quantity and Quality as Compared to Unit Mass Resolution Mass Spectrometry in High-Throughput Profiling Metabolomics. Metabolomics 4:2. https://doi.org/10.4172/2153-0769.1000132
11. Chen GC, Chai JC, Yu B et al (2020) Serum sphingolipids and incident diabetes in a US population with high diabetes burden: the Hispanic Community Health Study/Study of Latinos (HCHS/SOL). Am J Clin Nutr 112(1):57-65. https://doi.org/10.1093/ajcn/nqaa114
12. Zhang Y, Spitzer BW, Zhang Y et al (2025) Untargeted metabolome atlas for sleep-related phenotypes in the Hispanic community health study/study of Latinos. EBioMedicine 111:105507. https://doi.org/10.1016/j.ebiom.2024.105507
13. Dehaven CD, Evans AM, Dai H, Lawton KA (2010) Organization of GC/MS and LC/MS metabolomics data into chemical libraries. J Cheminform 2(1):9. https://doi.org/10.1186/1758-2946-2-9
14. Metabolon - Support | Portal - Experimental Procedures (2025). Available from https://www.metabolon.com/software/support/portal/experimental-procedures/. Accessed January 24, 2026
15. Inker LA, Eneanya ND, Coresh J et al (2021) New creatinine- and cystatin C-based equations to estimate GFR without race. N Engl J Med 385(19):1737-1749. https://doi.org/10.1056/NEJMoa2102953
